# Plastic packaging waste in Europe: Addressing methodological challenges in recording and reporting

**DOI:** 10.1177/0734242X221142192

**Published:** 2023-01-15

**Authors:** Celia Somlai, Craig Bullock, John Gallagher

**Affiliations:** 1 School of Architecture, Planning and Environmental Policy, University College Dublin, Dublin, Ireland; 2 Civil, Structural and Environmental Engineering, Trinity College Dublin, Dublin, Ireland

**Keywords:** Plastic packaging waste, plastic waste generation, waste analysis, placed on the market, waste statistics, circular economy

## Abstract

A reliable comparison of European Union (EU) Member States’ reporting of statistics on plastic packaging waste generation, recycling and recovery is necessary if there is to be a fair transition to a circular economy across the EU. It is a priority for there to be an improvement in the quality and validity of these statistics to assess each Member State’s performance in relation to EU targets. This article explores the quality of national reporting based on the two main approaches which are used to calculate packaging waste generation, namely ‘placed on the market’ and ‘waste analysis’. The findings outline how Member States apply a variety of approaches leading to different packaging waste statistics which makes reported data difficult to compare. Often, it is not clear what approach is applied in different counties. Factors such as freeriding, non-compliance and de minimis have represented the primary weaknesses in evaluating and reporting packaging waste statistics as producers have financial incentives for under-reporting. This article highlights the need to inform circular economy strategies by addressing the challenge of comparing data on packaging and plastic packaging waste generation across the EU.

## Introduction

The European Union (EU) has committed itself to creating a more circular and resource-efficient economy. Plastic reuse and recycling were identified as key priorities in the first EU Action Plan for a Circular Economy (EC (2015). As such, the European Strategy for Plastic in a Circular Economy (EC, 2018) outlined ambitious targets for reducing plastics consumption and increasing recycled content, aiming to have 55% reusable or recyclable plastic packaging by 2030.

For these closed material loop ambitions to be realized there needs to be a clear understanding of the amounts and types of waste, which includes having accurate estimates of current waste generation and the contribution made by packaging (Elgie et al., 2021). This requires a framework that permits analysis and comparison of different streams and types of packaging, particularly the complexity of plastic packaging (Matthews et al., 2021).

Globally, packaging and packaging waste management data is recorded using a variety of methods and approaches. These are usually unable to cover the entire waste stream and vary in their completeness (Eunomia, 2017). For the data that is available, methodologies are inconsistent and activities poorly recorded. Consequently, creating internationally comparable statistics on waste generation and management is challenging due to the variety of national waste management systems.

Recent studies of EU practice by Robaina et al. (2020) and Cimpan et al. (2021) highlight the complexity of plastic waste data analysis. Reliable and comprehensive waste data assists with setting future targets that will support legislation for environmental protection (Sahimaa et al., 2015). This is essential for monitoring waste management performance against national and EU targets (Brunner and Rechberger, 2016).

### European legislation on packaging waste

The management of packaging waste has been an integral part of European waste policy. From the first EU Directive 85/339/EEC adopted on Packaging and Packaging Waste to the subsequent Directive 94/62/EC that led to standardized national policies and ‘essential requirements’ for manufacturing practices and composition requirements, the ambition has been to prevent or reduce the impact of this waste on the environment. In 2018, the Directive (2018/852) was revised to include recyclability targets for packaging waste and consider how product design can improve reuse or recycling (European Union, 2018). The Single Use Plastics Directive (2019/904) and the updated Circular Economy Action Plan (COM/2020/98) have set a target for 90% of plastic bottles to be recycled by 2029, and that these be required to contain a minimum of 30% recycled plastic by 2030.

This Directive has obligated EU Member States to report all packaging waste data. The initial EU-15 have been reporting statistics since 1997 and the 10 countries who joined in 2004 have submitted data since 2005. Member States report their packaging waste data statistics in line with the most updated system of Commission Implementing Decision (EU) 2019/665, representing a revision of the original Directive 94/62/EC (EC, 2019). These include the reporting of packaging waste generation quantities, material and all forms of recycling, all forms of energy recovery, including incineration, and total recovery within or outside the originating Member State. New reporting guidance was prepared by Eurostat (Eurostat, 2022a). This includes the submission of mandatory information by national authorities on the material totals and types of different packaging within 18 months of the reference year. In addition, under the Own Resource requirements introduced in January 2021, Member States are now being asked to make a contribution to the EU budget based on estimates of non-recycled plastic packaging waste representing the difference between the quantifies of plastic waste generated and that which is recycled. It is intended that this requirement will provide an incentive to meet the packaging and recycling targets to achieve a circular economy as part of Europe’s Green Deal.

### Plastic packaging pathways

Of packaging materials, plastic is ubiquitous as it is a low-cost, strong, durable, lightweight, easily mouldable, water-resistant and bio-inert material. However, when it cannot be recycled or disposed of properly, plastic pollution can present a serious environmental problem. Marine plastic litter is considered one of our most pressing contemporary environmental challenges (Nielsen et al., 2019).

These negative impacts are compounded by plastic’s abundance as its use continues to grow, particularly for packaging. Between 1964 and 2020, the global production and consumption of plastics has increased more than 24 times, from 15 million tonnes (MT) to 367 MT.^1^ Only 9% of the 6300 MT of virgin plastic manufactured by 2015 has been recycled, with 12% incinerated and the remaining 79% accumulating in landfills or in the natural environment as litter (Geyer et al., 2017). At least 150 MT has accumulated in the oceans (Ocean Conservancy, 2015).

In 2019, Europe produced approximately 15% of the estimated 370 MT of global plastics. The European plastics industry, which produces raw material and undertakes plastic conversion and recycling, had a turnover of €350 billion in the same year (Plastics Europe, 2020). In addition to its low cost and versatility, a shift from cardboard or metal containers to convenient single-use plastic products has led to this large market share. Approximately 42% of all non-fibre plastic production globally (146 MT) is now used for packaging (Geyer et al., 2017) and there has been a 4.2% annual global increase in food and drink packaging placed on the market (PoM) since 2010 (Ketelsen et al., 2020).

How producers balance marketing, product protection, product preservation and reuse/recycling considerations has a large influence on trends in its use. Recently, producers have been keen to promote the sustainability of their products. In contrast, plastic pouches and composite packaging combining plastic with cardboard, foil or other materials have become popular due to consumer perceptions of convenience and of superior quality or freshness.^2^ Both products compound the segregation of packaging waste and indicate that producers have not yet felt obliged to respond to policies that aim for increased recyclability.

**Figure 1.**
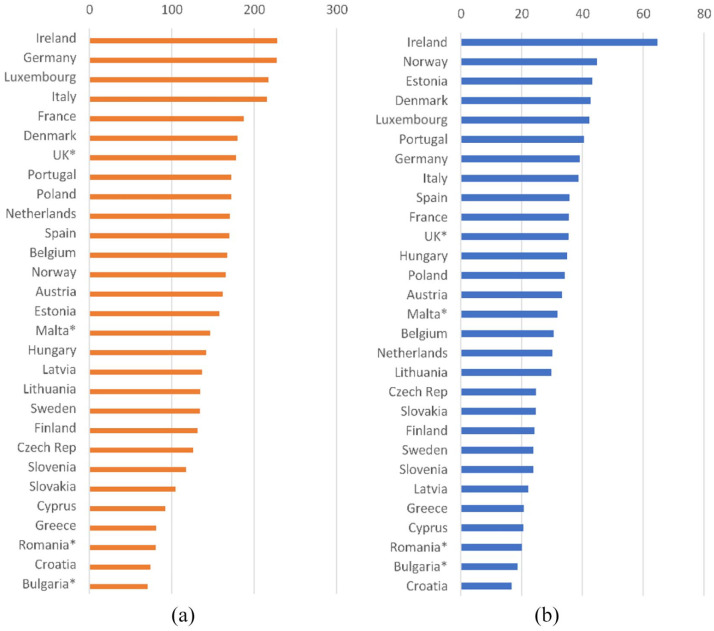
Kilograms per person of (a) packaging and (b) plastic packaging waste generation in Europe in 2019. Source: Eurostat env_waspac. (https://ec.europa.eu/eurostat/databrowser/view/env_waspac/default/table?lang=en Data c).

Packaging waste reached a record of 178 kg per capita in 2019 (Eurostat, 2022b). Plastic is the next most common packaging waste material (19.4%) after paper and cardboard (40.6%), and of concern given that the 15.4 MT of plastic waste generated in the EU-27 in 2019 represents an increase of 9.6% on 2008 (Figure 1). Official data indicates that Ireland reported the largest amount of plastic packaging waste generated per capita, at 65 kg, a figure that is 87% greater than the average for all EU Member States.

### Aim of the article

This article examines the impact of different reporting methods for plastic packaging waste generation and recycling in the EU. Ireland (in which the article’s authors are based) appears to produce the highest quantity of plastic packaging (Figure 1). It applies the ‘waste analysis’ (WA) method along with a small number of other EU Member States. Most other EU Member States apply the ‘PoM’ method or a mix of approaches. Therefore, this article evaluates these two main methods and compares their approaches and limitations. The article makes recommendations on a way forward to align these methods and improve reporting to support a harmonized European approach to the circular economy, although it is based on data available in 2019 since when some Member States may have corrected data retrospectively or modified their data collection methods in response to the latest Eurostat reporting guidance (Eurostat, 2022a).

## Methodologies for collecting of packaging waste statistics

A variety of methods have been developed to describe the amount and composition of waste generated. There is no single international standard, but guidance is provided by EU and national agencies. The three main methods to characterize municipal solid waste (MSW) are (i) direct WA (or waste composition analysis), the traditional approach that comprehensively examines individual samples of post-consumption waste; (ii) market product analysis (or the ‘PoM’ approach) is based on national statistics on production and sales provided by producers before packaging enters the market; and (iii) waste product analysis which characterizes products after incineration, but which is outside the scope of this article (Brunner and Ernst, 1986).

Article 2(2) of the EC Decision (2 March 2005)^3^ relating to Directive 94/62/EC states, ‘Packaging waste generated in a Member State may be deemed to be equal to the amount of packaging PoM in the same year within the Member State’. This signals acceptance of the two main methodologies, WA and PoM, to estimate the quantity of packaging waste generated.

The reliability of both methods is dependent on the quality of the data collected or sampled. Eurostat guidelines (2022a) propose that WA involve numerous samples of equal weight or volume, broken down into different waste categories, across different time of year, and accounting for factors such as settlement structure, household characteristics, socio-economic factors, and the waste services or charging schemes in operation. It also proposes that PoM data be collected directly from producers or from independent consulting companies, or as submitted to national or regional authorities, or by producer responsibility organizations (PROs). The quality of the data can be assessed at different stages of reporting by different stakeholders (e.g. PROs, statistical offices, national authorities, independent auditors) and supported with cross-checks, inspections, or audits.

**Figure 2.**
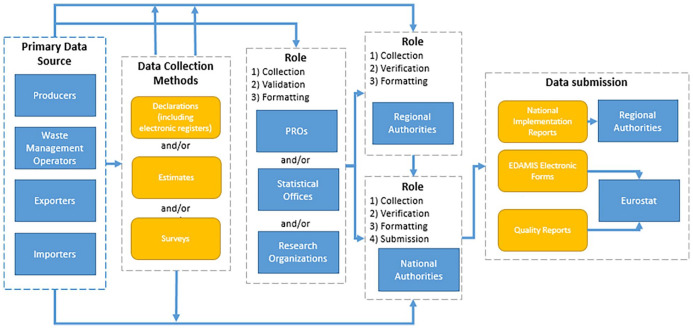
Overview of reporting on packaging and packaging waste. Source: Eunomia (2017).

Until recently, EU Member States were asked to accompany their annual waste statistics with ‘quality reports’ to inform the Commission of calculations, sources of information, and reliability of data. However, only a minority of reports were submitted on time and no specifications were provided to ensure standardized reporting. This made it challenging to obtain information on the particular methodology used by each country. Since 2021, quality reports are now more detailed and are obligatory (for the reference year 2019 onwards). They show that Member States use different methodologies, typically based on historic approaches, including estimates based on trade statistics and/or waste management statistics. Figure 2 shows the main steps and stakeholders involved in reporting packaging and packaging waste (Eunomia, 2017). Table 1 provides an overview of the different methods, or mix of methods, used by EU Member States.

**Table 1.** Overview of source and methodology for reference year 2017 to calculate data for packaging waste generated in different Member States.

Member state	Placed on the market				Waste analysis
	EPR Scheme	Industry declaration	Production and foreign trade	Questionnaires	
AT	Y	N	N	Y	Y
BE	Y	Y	N	Y	N
CY	Y	Y	N	N	N
CZ	Y	Y	N	N	N
DE	Y	Y	Y	Y	N
DK	N	N	Y	N	N
EE	N	N	N	Y	Y
ES	N	Y	N	N	N
FI	Y	N	N	N	N
HR	Y	N	N	N	N
HU	Y	N	N	Y	Y
NL	Y	Y	N	Y	N
PT	Y	N	N	N	Y
SE	Y	N	N	Y	N
SK	Y	N	N	Y	N
UK	Y	Y	Y	Y	Y

Source: Eurostat.

EPR: extended producer responsibility.

### Placed on the market

The PoM approach, developed in the early 1970s, is more prevalent in EU Member States and requires information on the production and consumption of different economic actors. Data collection has evolved over time, while having needed a clearer distinction between data that is declared or data which is calculated. PROs emerged in the 1990s and collect and report data on behalf of manufacturers and retailers. Adjustments are applied to account for imports, exports and cross border sales (Brunner and Rechberger, 2016). Although not verified, official import and export data are obtained from trader self-declarations (Chung, 2008). As such, data collection varies among Member States based on dependence of: (a) extended producer responsibility (EPR) schemes, (b) statistical offices, and (c) industry declarations.

#### EPR schemes

Sixteen Member States report packaging through EPR schemes which exist in almost all (25) of the EU-27 (IEEP, 2017) for self-reporting the quantity of packaging they are producing or selling. The responsibility of producers is extended to the post-consumer stage of a product’s life, including take-back, recycling and final disposal (Lindhqvist, 2000). All schemes have some basic fee modulation based on different packaging materials. However, these measures have failed to discourage the use of very light materials such as plastics. There is, however, increasing use of advanced eco-modulation under which fees are low when the packaging is reusable, and high when it is non-sortable, non-recyclable or made of composite materials. Article 8a(4)(c) of the Waste Framework Directive (WFD) will increase fee modulation and reduce the use of non-recyclable packaging. However, EPR frameworks still need to be more harmonized and transparent to improve data quality (Leal Filho et al., 2019).

#### Statistical offices – Production and trade statistics

Data from production and foreign trade statistics, along with some surveys and electronic registries, are used by at least five Member States to estimate packaging waste data. For example, Denmark combines general statistics on empty packaging production with statistics on foreign trade in filled packaging. Calculations are based the composition and packaging assumptions for almost 9000 different product groups, including an assumption that import and export products are similarly packed. This sophisticated method has inevitable uncertainty given the assumptions associated with so many product groups.

#### Industry declarations

Self-declarations by industry are used by around 13 Member States while questionnaires or statistical compilations and other sources of data (from consultancies or local authorities) are used by at least another 13 Member States. This ranges from Spain, where only industrial declarations are used, to the Netherlands which applies a hybrid system using PoM data compiled from the country’s EPR scheme plus industry declarations and questionnaires, or Germany, where the Association of Packaging Market Research compiles data collected from trade statistics, industry declarations and numerous individual studies and surveys. Additional means may be applied to refine estimates for particular businesses, including expert advice or spot checks. There are, however, few examples of businesses being penalized for misreporting (Eunomia, 2018).

### Waste analysis

WA was developed in the 1960s and 1970s to classify the quantities and qualities of waste, that is, types of materials (Brunner and Rechberger, 2016). In Europe, it is used primarily by small Member States (Ireland, Estonia, Luxembourg and Austria) in which many products are imported from abroad, but partly also by Portugal, Hungary and the United Kingdom. Also known as the ‘sample-and-sort’ method, it involves taking waste samples at point-of-generation (i.e. household or business) or at a waste-processing facility. In principle, WA should provide an accurate waste generated measurement as it captures all packaging, including from on-line sales, free-riders and small companies.

The characterization of waste material composition typically consists of four phases: (i) analysis planning and design, (ii) waste sampling, (iii) component waste category sorting (e.g. paper, plastic, etc.) and (iv) data evaluation and interpretation. This analysis provides information for various purposes in addition to reporting, including local waste management, planning and recycling campaigns (Sahimaa et al., 2015). Eunomia (2017) has recommended obligatory periodic sampling and statistically accurate waste composition analysis be required of Member States to establish reliable statistics of plastic packaging waste. This would need best practice guidance and a common methodology for all EU Member States.

## Challenges and limitations

In principle, the amount of packaging waste calculated using WA and PoM should be equal due to the short lifespan of most packaging. However, in practice, there are clear discrepancies, as well as the absence of a common approach to data collection. This has implications for the comparability of figures across the EU.

### Limitations of PoM

The variety of methodologies, data sources and level of validation for measuring plastic packaging PoM is problematic. Inaccuracies arise from the following:

- irregular or incomplete reporting, for example, over-reliance on occasional studies or surveys;
- double-counting due to uncertainty over who in the production chain is responsible for reporting;
- incomplete industry data, for example, where sample sizes are not representative, or when incorrect calculation has been applied;
- declaration of predominant packaging material to the exclusion of others packaging components such a plastic lids or bottle tops; and
- lack of comprehensive import and export data, including that by third parties.

#### De minimis thresholds

De minimis applies to smaller producers who are not obligated to provide data. New Eurostat guidelines now recommend that small producers be included in EPR based on a turnover threshold for which regular national reviews are proposed to ensure consistency with packaging weight (EC, 2020). Comparisons for former years were complicated by the varying reporting thresholds and level of thoroughness applied by Member States (Table 2), making for incomplete coverage of plastic packaging generation. Indeed, a likely underestimation is acknowledged by the Commission (EC, 2020).

**Table 2.** Examples of de minimis for the obligatory declaration of plastic packaging placed on the market.

Country	Plastic packaging (kg)	Turnover
Latvia^1^	300 (total)	NA
Germany^2^	30,000	NA
Austria^3^	100	€730,000
Czechia^4^	300	CZK 4,500,000
Netherlands^5^	50,000 (total)	NA
The United Kingdom^6^	50,000 (total)	£2 m
Portugal^7^	Not in place	Not in place
Ireland^8^	10,000	€1,000,000

1 https://likumi.lv/ta/id/219851-noteikumi-par-izlietota-iepakojuma-regeneracijas-procentualo-apjomu-registresanas-unzinojumu-sniegsanas-kartibu-un-iepakojuma (accessed 22 November 2022).

2 https://www.bgbl.de/xaver/bgbl/start.xav?startbk=Bundesanzeiger_BGBl&jumpTo=bgbl117s2234.pdf (accessed 22 November 2022).

3 https://www.ris.bka.gv.at/GeltendeFassung.wxe?Abfrage=Bundesnormen&Gesetzesnummer=20008902 (accessed 22 November 2022).

4 https://www.mzp.cz/C125750E003B698B/en/packaging_legislation/$FILE/OODP-Act_on_Packaging_No_477_2001-20110111.pdf (accessed 22 November 2022).

5 https://zoek.officielebekendmakingen.nl/stcrt-2017-75133.html (accessed 22 November 2022).

6 https://consult.defra.gov.uk/extended-producer-responsibility/extended-producer-responsibility-for-packaging/supporting_documents/23.03.21%20EPR%20Consultation.pdf (accessed 22 November 2022).

7 Email confirmation from the Portuguese Environmental Protection Agency.

8 https://www.irishstatutebook.ie/eli/2014/si/282/made/en/print (accessed 22 November 2022).

#### Extended producer responsibility

The different EPR arrangements and responsibilities for waste data collection lead to poor comparability. There is a lack of harmonization and transparency across the EU and no common approach to the collection of data (OECD, 2014; Zero Waste Europe, 2015). For example, in Sweden, all packaging reporting is based on information provided by PROs to the Swedish Emissions Data Consortium. For other Member States, it has often been unclear what methodology has been applied to collect data or for quality assurance. The characteristics of PROs vary considerably. Also, 12 Member States (41%) have one EPR scheme, whereas 9 others (33%), such as Portugal, have competing schemes.

There is also currently no clear information on what different Member States define as packaging waste or how this waste is related to its source. This could include separable small components such as lids or bottle tops. As each scheme applies different waste types, or focuses on different packaging waste streams (household, commercial or industrial), consistent declarations for packaging are unlikely. EPR schemes extend only to municipal packaging in Portugal; to household and equivalent packaging in France and Germany, household and commercial packaging in Belgium; and exemption of tertiary (transport) packaging in the Netherlands.

In all schemes, there is an incentive for companies to report lower quantities of packaging to minimize fee obligations. This incentive is affected by the responsibilities of PROs, especially where they are competing, and the degree to which waste management costs are passed on to members. Some PROs accept full responsibility for collection and recycling as in Denmark, while for others, the responsibility is financial in that members’ fees are used to fund take-back or recycling. It is for this reason that the Commission is seeking harmonization to fairly reflect the costs of recycling different plastic packaging materials.

Reporting the lowest possible plastic packaging waste generation and as high as possible a recycling rate is beneficial for all stakeholders. Free riders are at the extreme of this spectrum as they place plastic packaging on the market, but often do not report data or take responsibility for the costs of collection and treatment. Articles 8a(1)(d) and 8a(5) of the WFD aim for the equal treatment of producers, including the avoidance of freeriding. However, there is a continuing trend towards more online and cross border sales, but with this packaging being unaccounted for by national packaging compliance schemes.

Online sales platforms are a major contributor to freeriding, for example, with electrical and electronic equipment (EEE) where 10–30% are not registered, possibly amounting to almost 9% of all EEE sales in the EU (OECD, 2018). Cross border trade exacerbates freeriding as some packaged items are purchased in one country, but with the packaging being disposed of in another. Unbalanced, single directional flows of goods, including packaging, likely occur where different tax rates apply to packaged good prices between neighbouring Member States, for example, Denmark and Germany (Eunomia, 2018).

### Limitations of WA

The sampling procedure used for WA has a profound effect on the quality of the waste composition data, including estimates of the proportion and type of packaging. The Solid Waste Analysis tool project^4^ delivered a European standard method for the characterization of MSW, recommending complex best practice methods for sampling and sorting of household waste. In practice, different methods continue to be used in Europe and globally (Edjabou et al., 2015; Sahimaa et al., 2015).

To ensure accurate compositional sampling, it is necessary to take frequent and consistent samples of adequate size (Sahimaa et al., 2015). All national systems involve sampling at source or from vehicle loads brought to waste transfer or treatment facilities. Sieving can be a source of error as the proportion of unidentifiable small items, or *fines*, can vary. In addition, to capture all the packaging waste in MSW, different waste composition analysis is needed, including a detailed analysis of separately collected recyclates and of other packaging that ends up in residual solid waste, including material from drop-off facilities, street bins, private disposal and as large bulky refuse (Expra, 2015). Assumptions need to be made about the proportion of packaging waste ending up as litter or which is unmanaged or dumped illegally.

There are also different views on sample size in the literature (Nordtest, 1995). The objective is to achieve a correct assessment of the average waste composition with 10% accuracy and a confidence level of 95%. The Solid Waste Analysis tool recommends a 45 m^3^ and 100 m^3^ waste bin volume as the minimum sample size for household and non-household waste accordingly. Assumptions with regard to local and seasonal variations in waste generation need to be applied too and each sample should cover at least one full week of waste. As a rule of thumb, a minimum number of samples for characterisation is 10 if the sample size is 100 kg or larger. For a single stratum (waste course/type), a minimum of 5 × 100 kg is argued to provide a rough, but reasonable result (Dahlen and Lagerkvist, 2008).

The most crucial choices for compositional sampling are stratification (i.e. the choice of a relevant number of waste sources and types), sampling procedure, sample size and number and the type and number of waste component categories. An essential stratification occurs between household and other waste, but other important strata include urban/rural, residential structure (e.g. single-family homes, apartment blocks), separation at source (e.g. number of bin types), season, tourism locations, socio-economic differences or availability of bring centres/civic amenity sites (EC, 2004; Edjabou et al., 2015; Sahimaa et al., 2015). It is assumed that variation within a common stratum will be less the lower the overall population variance and that, subject to the number of strata selected, fewer samples are needed overall (EC, 2004).

The results from waste composition studies cannot be reliably compared if there are significant differences in the definition of these waste categories. Data is generally available for the main polymer types and main sources of waste. However, specific breakdowns of packaging by source or sector are few, including estimates of the proportion of food and non-food packaging. This makes it more difficult to resolve issues of unsustainable packaging at source. The characteristics of waste also change over time as new packaging products are introduced to the market. For example, it’s the constituent materials of composite packaging can be challenging to determine as they are often laminated together. Both waste characterization and data derived from trade data will typically fail to keep pace with these changing trends (Brunner and Rechberger, 2016).

More fundamentally, most of the waste characterization studies report the arithmetic mean and standard deviation of waste fraction compositions, ignoring the natural structure of compositional data. However, compositional data obscures the fact that different variables in the dataset are inherently dependent on each other. As traditional statistical operations are based on the assumption of variable independence, computing arithmetic means, standard deviations and correlation coefficients for material fraction compositions may generate erroneous and biased results. Instead, Edjabou et al. (2017) recommend that fractional waste composition data should be transformed appropriately prior to statistical analysis.

#### Contamination and moisture

Packaging PoM is dry and free of extraneous material and contaminants. However, potential sources of error in WA arise from estimates of moisture (humidity) and contamination. Moisture adds significantly to the weight of paper and board, but and to plastics too. Levels vary across Europe due to climate and season. Light materials such as paper or plastics can also be heavily contaminated by labels, glues, inks and food residue. Member States are required to ensure that WA samples are dry and clean, but extrapolation requires a correction factor that accounts for the different strata sampled. Eurostat guidance (2022a) proposes that adjustments should be made where moisture exceeds the ‘natural humidity’ that can be expected for a packaging type in any particular Member State.

A study by WRAP (2015) estimated non-target materials as accounting for 10% by weight of MSW. Contamination depends also on packaging type with other studies reporting up to 65% for non-recoverable plastic packaging, 39% for drinks cartons and 41% for plastic bags and films, with lower comparable waste statistics for the contents of recycling bins, but varying and inconsistent results for two bin systems and three bin systems that include a food waste bin (CTC, 2018). Contamination also depends on whether recyclables are collected from household or non-household waste, whether packaging is collected from MSW or from sorted mixed dry recyclables, and on the number of bins available to a household for segregation. It is therefore essential that correction factors are applied to account for differences in weight due to contamination, but the variation makes it difficult to arrive at reliable and consistent adjustments. Eunomia (2018) recommend a correction factor of 25%, but actual correction factors vary between Member States, being 18–19% in Austria and up to 35% for mixed waste in Luxemburg. In some Member States, correction factors are not added at all.

#### Other sources of error

Other source of error can arise from a failure to account for non-household flows of material. The proportion represented by street litter will vary from region to region or country. Street littler is likely to have a high level of moisture and food waste. There may also be a need to account for the removal from the waste stream of recyclables collected by non-profit organizations, material set aside for home composting, and also the burning of packaging waste in back yards. The inclusion of commercial waste can introduce higher variation as waste generation by businesses and public institutes is less homogenous than household waste.

#### Practical considerations

While data quality will increase with increased sample size, greater sampling frequency and the number of strata, so too will the cost and labour intensity of sampling. The composition and properties of MSW change constantly. It is also necessary to account for seasonal variation (Chung, 2008) and to sample with sufficient frequency to ensure that longer term trends are identified. Data is challenging to compare over time and between regions in larger Member States where consumer behaviour and waste management vary from region to region. In practice, however, the expense of thorough sampling means that Member States relying on WA are tempted to sample only very infrequently.

## Comparing packaging waste data of Member States using PoM and WA

In Europe, Member States are encouraged to use either WA or PoM, but no country applies both methods equally. Rather, they are prompted to also use alternative means for verification and now to cross-check between the two approaches on an annual basis. Due to the expense, the frequency of sampling for WA varies between Member States. Figure 1 showed that Ireland ranks high on the table for waste generation based on its WA data, a position contested by the Irish authorities. However, if a 20% random sampling error were present (as is quite possible given the difficulty of achieving a representative sample for waste), and assuming there to be an overestimate of the amounts generated, then the estimate of per capita plastic packaging waste could be reduced to 46 kg per capita, a level closer to those of Estonia and Luxembourg. If a 35% sampling error is present, this would lower the amount to 38 kg per capita, around the same level as the United Kingdom, France, Italy and Spain.^5^

**Figure 3.**
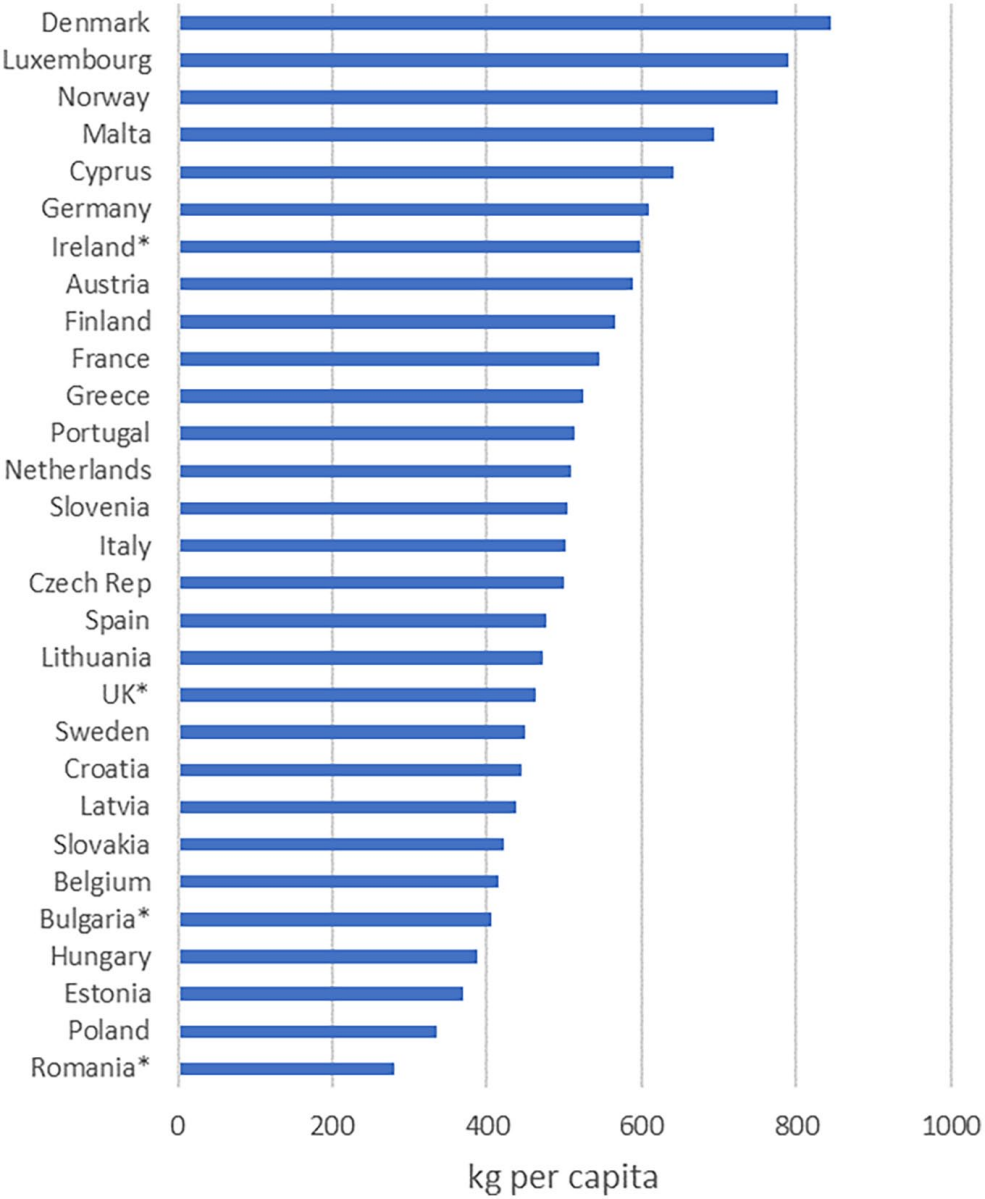
Municipal solid waste generated in 2019. Source: Eurostat (MS using WA (Estonia, Luxembourg, Austria, Portugal and Hungary). *2018

As a check, we can compare the packaging and plastic packaging waste generated (Figure 1) with MSW per capita shown in Figure 3. A ratio of MSW generated and packaging data from WA involves the comparison of related waste streams and is likely to be more accurate than a comparison with PoM. These data show that Ireland’s waste generation is similar to Member States such as Austria and France, and not too far from the former EU-28 average, while still being seventh in the rankings. In terms of the proportion of MSW which is packaging shown in Table 3, Ireland is similar to the United Kingdom which mostly relies on PoM, although plastic packaging is second only to Estonia which also uses the WA approach. In per capita terms, this indicates that, while the amount of plastic packaging waste generated may indeed be high, Ireland is less likely to be near the top of the rankings for packaging waste.

**Table 3.** Packaging and plastic packaging – A proportion of MSW 2019.

Country	Packaging (%)	Plastic packaging (%)
Higher in table		
Estonia	58.9	11.7
Poland	49.3	10.2
Hungary	43.3	9.0
Ireland*	38.1	10.8
United Kingdom*	36.7	7.7
Lower in table		
Malta	22.7	4.6
Bulgaria*	18.2	4.6
Greece	17.6	4.0
Croatia	15.9	3.8
Cyprus	12.6	3.2

* 2018.

Furthermore, while Member States such as Ireland who are using the WA approach might be overestimating the amount of plastic packaging waste generated, it is at least equally possible that some Member States reporting low levels of plastic packaging waste generated could be underestimating. These countries’ MSW volumes can be expected to be low given that the amount of municipal and plastic waste generation is proportional to average mean living standards (Wertz, 1976). However, there are numerous other factors which can influence waste generation including sociocultural factors influencing consumption patterns, as well as climate, recycling facilities, deposit return systems, sustainability awareness, and social and public attitudes. The possibility of underestimation is evident by triangulating reported MSW generation, packaging waste generation and packaging recycling rates. By combining these three datasets with an understanding of general packaging waste trends across the EU, it is possible to highlight which Member States might be under-reporting.

## Conclusions and proposals to improve reporting

This study highlights a lack of harmonization in the methods adopted for reporting. Packaging and plastic packaging waste management is improving in Europe. The export of plastic waste to developing countries was banned in 2021, but a large share continues to go to incineration or to landfill. A circular economy transition presents policy and planning challenges, requiring reliable and harmonized approaches to measuring plastic packaging waste as evidence to drive change.

The predominant ‘PoM’ approach commonly *underestimates* packaging waste generation. Despite clearer data collection guidelines, less developed EU Member States declare questionably low levels of packaging as a proportion of MSW. More developed Member States apply inconsistent methods and have a reliance on incomplete EPR reporting. PROs also have an incentive to under-report as it reduces their fee obligations.

PoM assumes all packaging waste to be clean, dry and uncontaminated. In contrast, the WA method used by a minority of Member States must account for packaging waste which is contaminated by non-packaging materials or has a higher moisture content. The method also has the virtue of providing valuable data to inform national governments’ waste management and recycling strategies. It is costly to apply, however, demanding thorough and frequent sampling with the best composition analysis applying to packaging waste estimates taken over several years.

The 2022 Eurostat guidelines will go some way to improving the quality of the reporting of plastic packaging waste, but further harmonization will take time given long-standing national conventions on reporting. The issue of de minimis is being addressed to ensure that every producer reports their turnover as an indicator of packaging waste PoM. As the opportunity for freeriding and non-compliance, especially due to online sales, is a weakness of PoM data, importers and e-commerce platforms must be required to register and report their packaging statistics. For WA, statistical comparison will benefit from more consistent sampling and treatment of contamination, and from regular surveys of the different moisture levels of packaging when PoM and at collection.

In all cases, there is a benefit in all Member States applying the alternative method of data collection to help verify and explain differences in nationally reported tonnages. A clearer definition of what constitutes packaging waste in different Member States is needed. In addition, further progress on the eco-modulation of EPR fees would encourage greater recyclability, including of the increasing amount of composite packaging.

## Notes

1.
https://www.statista.com/statistics/282732/global-production-of-plastics-since-1950/

2. GlobalData (http://www.globaldata.com/)

3. Commission Decision (22 March 2005) Establishing the Formats Relating to the Database System Pursuant to Directive 94/62/EC of the European Parliament and of the Council on packaging and Packaging Waste (Document Number C(2005) 854) (2005/270/EC).

4.
https://cordis.europa.eu/project/id/EVK4-CT-2000-00030

5. WRAP (2015) Guidance on the Methodology for Waste Composition Analysis for local authorities commissioning waste composition analysis of municipal waste, Report June 2015, https://www.zerowastescotland.org.uk/sites/default/files/WCAMethodology_Jun15.pdf
